# Study design of a randomised, placebo-controlled trial of nintedanib in children and adolescents with fibrosing interstitial lung disease

**DOI:** 10.1183/23120541.00805-2020

**Published:** 2021-06-21

**Authors:** Robin Deterding, Matthias Griese, Gail Deutsch, David Warburton, Emily M. DeBoer, Steven Cunningham, Annick Clement, Nicolaus Schwerk, Kevin R. Flaherty, Kevin K. Brown, Florian Voss, Ulrike Schmid, Rozsa Schlenker-Herceg, Daniela Verri, Mihaela Dumistracel, Marilisa Schiwek, Susanne Stowasser, Kay Tetzlaff, Emmanuelle Clerisme-Beaty, Lisa R. Young

**Affiliations:** 1 Section of Pediatric Pulmonary and Sleep Medicine, Dept of Pediatrics, University of Colorado Denver, Denver, CO, USA; 2 The Children's Hospital Colorado, Aurora, CO, USA; 3 Hauner Children's Hospital, Ludwig Maximilians University, German Center for Lung Research (DZL), Munich, Germany; 4 Dept of Pathology, University of Washington School of Medicine, Seattle, WA, USA; 5 Seattle Children's Hospital, Seattle, WA, USA; 6 Children's Hospital Los Angeles, Los Angeles, CA, USA; 7 Keck School of Medicine, University of Southern California, Los Angeles, CA, USA; 8 Centre for Inflammation Research, University of Edinburgh, Edinburgh, UK; 9 Pediatric Pulmonary Dept, Trousseau Hospital, AP-HP Sorbonne University, Paris, France; 10 Clinic for Pediatric Pulmonology, Allergology and Neonatology, Hannover Medical School, Hannover, Germany; 11 Division of Pulmonary and Critical Care Medicine, University of Michigan, Ann Arbor, MI, USA; 12 Dept of Medicine, National Jewish Health, Denver, CO, USA; 13 Boehringer Ingelheim Pharma GmbH & Co. KG, Ingelheim am Rhein, Germany; 14 Boehringer Ingelheim Pharmaceuticals Inc., Ridgefield, CT, USA; 15 Boehringer Ingelheim Italia S.p.A., Milan, Italy; 16 Boehringer Ingelheim International GmbH, Ingelheim am Rhein, Germany; 17 Sports Medicine Dept, University Hospital of Tuebingen, Tuebingen, Germany; 18 Division of Pulmonary and Sleep Medicine, The Children's Hospital of Philadelphia, Philadelphia, PA, USA; 19 These authors contributed equally

## Abstract

Childhood interstitial lung disease (chILD) comprises >200 rare respiratory disorders, with no currently approved therapies and variable prognosis. Nintedanib reduces the rate of forced vital capacity (FVC) decline in adults with progressive fibrosing interstitial lung diseases (ILDs). We present the design of a multicentre, prospective, double-blind, randomised, placebo-controlled clinical trial of nintedanib in patients with fibrosing chILD (1199-0337 or InPedILD; ClinicalTrials.gov: NCT04093024).

Male or female children and adolescents aged 6–17 years (≥30; including ≥20 adolescents aged 12–17 years) with clinically significant fibrosing ILD will be randomised 2:1 to receive oral nintedanib or placebo on top of standard of care for 24 weeks (double-blind), followed by variable-duration nintedanib (open-label). Nintedanib dosing will be based on body weight-dependent allometric scaling, with single-step dose reductions permitted to manage adverse events. Eligible patients will have evidence of fibrosis on high-resolution computed tomography (within 12 months of their first screening visit), FVC ≥25% predicted, and clinically significant disease (Fan score of ≥3 or evidence of clinical progression over time). Patients with underlying chronic liver disease, significant pulmonary arterial hypertension, cardiovascular disease, or increased bleeding risk are ineligible. The primary endpoints are pharmacokinetics and the proportion of patients with treatment-emergent adverse events at week 24. Secondary endpoints include change in FVC% predicted from baseline, Pediatric Quality of Life Questionnaire, oxygen saturation, and 6-min walk distance at weeks 24 and 52. Additional efficacy and safety endpoints will be collected to explore long-term effects.

## Abstract 2

**We describe the design of #InPedILD, a study of 24 weeks’ nintedanib or placebo on top of standard of care, followed by variable-duration open-label nintedanib in children with interstitial lung disease (ClinicalTrials.gov NCT04093024) #PedILD** https://bit.ly/3tC1a7P

## Introduction

Childhood interstitial lung disease (chILD) comprises >200 rare heterogeneous respiratory disorders that can affect infants, children and adolescents [1, 2]. The prevalence (1.5–3.8 cases per million [3–5]) and incidence (1.3 cases per million children [6]) of chILD may vary across different studies/analyses [1] depending on study design. chILD includes disorders that occur in adults as well as those unique to children, such as neuroendocrine cell hyperplasia of infancy and diseases attributed to genetic conditions and developmental processes [7]. Fibrosing forms of interstitial lung disease (ILD) involve an injurious process that can occur in both children and adults [1, 8]. It is not clear, however, whether the mechanism of fibrosis in the adult lung is similar to fibrosis in children who have ongoing alveolarisation [1].

Though fibrosing ILD in children has not been extensively studied and characterised, underlying conditions or contributing factors include surfactant dysfunction disorders such as mutations in *SFTPC*, *ABCA3* and *NKX2.1*, connective tissue disease-related ILD, and radiation- or drug-induced fibrosis [1, 7, 9]. Similar to adults, subgroups of patients with fibrosing chILD exhibit a progressive phenotype characterised by worsening symptoms, lung function decline and increased morbidity [1, 10]. There are no approved therapies for ILD treatment in children and, based on anecdotal evidence, the current standard of care comprises the empiric use of systemic steroids, other (steroid-sparing) immunosuppressants, hydroxychloroquine or azithromycin [1, 11, 12].

The tyrosine kinase inhibitor nintedanib potently blocks receptor and non-receptor tyrosine kinases that are implicated in the initiation and progression of pulmonary fibrosis, such as vascular endothelial growth factor (VEGF) receptors, platelet-derived growth factor receptors, fibroblast growth factor receptor kinase activity and Src family tyrosine kinases (*e.g.* Lck, Lyn and Flt-3) [13–15]. The antifibrotic effects of nintedanib have been demonstrated in various animal models of lung fibrosis resembling features of idiopathic pulmonary fibrosis (IPF) [16, 17], as well as in systemic sclerosis-associated ILD (SSc-ILD) [18] and rheumatoid arthritis-associated ILD [19]. Nintedanib treatment also decreased lung inflammation, granuloma formation and fibrosis in an animal model of silica-induced lung fibrosis [20] and reduced airway inflammation and remodelling following chronic allergic stimulation in ovalbumin-sensitised mice [21]. In animal models, nintedanib had an effect on tooth development and epiphyseal growth [22].

The benefits of nintedanib have been investigated in several fibrosing ILDs in adults, including IPF [23], SSc-ILD [24] and other progressive fibrosing ILDs (also described as chronic fibrosing ILDs with a progressive phenotype) [25]. Nintedanib is approved for the treatment of IPF, SSc-ILD and chronic fibrosing ILDs with a progressive phenotype in several countries [26, 27]. Across the clinical trial programme in adults, nintedanib is associated with a consistent and clinically meaningful slowing of the progressive decline in lung function as measured by forced vital capacity (FVC) over 52 weeks [23–25]. The most commonly reported adverse events have been gastrointestinal disorders, including diarrhoea, which were mostly mild or of moderate intensity and amenable to treatment [23–25]. Liver enzymes were also elevated in the nintedanib arms *versus* placebo [23–25].

To date, there have been no clinical trials of antifibrotic agents in chILD. Based on its mode of action, preclinical effects in animal models of ILD, and clinical benefit in various progressive fibrosing ILDs in adults, the use of nintedanib treatment for children and adolescents with fibrosing ILD is compelling. Although it is not currently feasible to conduct a fully powered clinical trial of efficacy in this patient population, a clinical study evaluating the pharmacokinetics and safety of nintedanib in children and adolescents (6–17 years old) with clinically significant fibrosing ILD was designed. The primary objective of this randomised, placebo-controlled clinical trial (1199-0337 or InPedILD; ClinicalTrials.gov: NCT04093024) is to inform the dosing and safety of nintedanib in this patient population. Efficacy assessment is also planned to explore the potential clinical benefit of nintedanib treatment in fibrosing chILD.

## Methods

### Trial design

This study is a multicentre, multinational, prospective, randomised, placebo-controlled clinical trial of nintedanib on top of standard of care for 24 weeks (double-blind), followed by variable-duration nintedanib (open-label) in children and adolescents with clinically significant fibrosing ILD. Patient recruitment is expected in ∼24 countries and 70 sites (∼1 patient screened per site).

**FIGURE 1:**
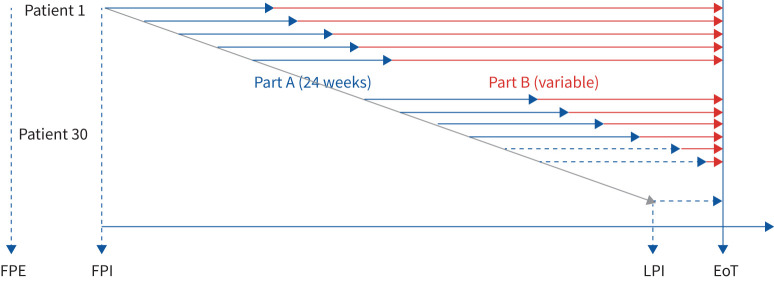
Study design. EoT: end of treatment; FPE: first patient enrolled; FPI: first patient in; LPI: last patient in.

Patients will undergo a 4-week screening period (visit 1 to visit 2). At visit 2, patients meeting the eligibility criteria will be randomised to enter the study treatment period (comprising part A and part B) (figure 1). During part A, patients will be randomised (2:1) to receive blinded treatment (nintedanib or placebo) for 24 weeks. Patients will receive either oral nintedanib or placebo (twice daily) on top of standard of care, with starting doses (50 mg, 75 mg, 100 mg or 150 mg twice daily) based on patient weight using allometric scaling. Following completion of part A (visit 6), patients will receive open-label nintedanib (part B) and will remain on treatment until the end of the study or discontinuation (variable from patient to patient).

The study will end when ≥30 patients (including ≥20 adolescents aged 12–17 years) have completed pharmacokinetic sampling at 26 weeks or have prematurely discontinued the trial. Patients who complete the per-protocol treatment period will be offered participation in a separate open-label extension trial, if supported by the benefit–risk assessment performed at the end of the double-blind period. Following the treatment period or after early treatment discontinuation, patients will enter a 4-week follow-up period, unless they roll over into the open-label extension trial.

### Participants

**FIGURE 2:**
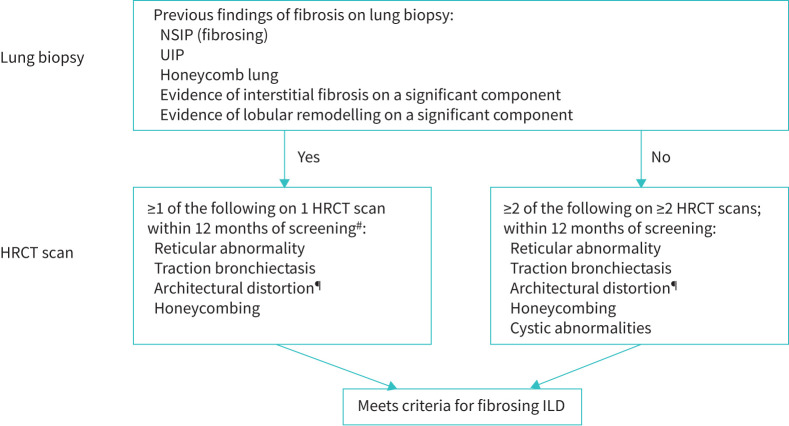
Inclusion criteria for fibrosing interstitial lung disease (ILD). Evidence of fibrosing ILD will be confirmed by central review (lung biopsy and high-resolution computed tomography (HRCT)). NSIP: non-specific interstitial pneumonia; UIP: usual interstitial pneumonia. ^#^: Coexisting cystic abnormalities or ground-glass opacity are acceptable; however, coexisting multifocal non-fibrotic, non-dependent consolidations (*e.g.* organising pneumonia, infection) will not be permitted. ^¶^: With or without ground-glass opacification.

Eligible children and adolescents (aged 6–17 years) will have clinically significant fibrosing ILD with fibrosis on lung biopsy or high-resolution computed tomography (HRCT) based on central review by an independent reviewer. Due to the lack of published guidelines validating imaging features of fibrosis in children, imaging criteria established by expert consensus will be used to confirm eligibility and ensure consistency (figure 2; Supplementary Methods).

Table 1 details eligibility criteria. Patients with underlying chronic liver disease [28], clinically significant pulmonary arterial hypertension [29], cardiovascular disease [30] or increased risk of bleeding [31] are ineligible. Patients who have previously received nintedanib or another investigational therapy (within 1 month or 5 half-lives) are ineligible. Potential diagnoses likely associated with lung fibrosis include, but are not limited to: surfactant protein deficiency (*SFTPC*, *ABCA3*, *NKX2.1* mutations); chronic hypersensitivity pneumonitis; toxic, radiation- and drug-induced pneumonitis; post-haematopoietic stem cell transplant fibrosis; and connective tissue disease-related disorders such as juvenile rheumatoid arthritis, juvenile idiopathic arthritis, SSc, dermatomyositis/polymyositis, mixed connective tissue disease or sarcoidosis.

**TABLE 1:** Full inclusion and exclusion criteria

**Inclusion criteria**
Male or female children and adolescents aged 6–17 years old at visit 2
Written informed consent and assent (where applicable) prior to admission to the trial
Female subjects of childbearing potential must confirm that sexual abstinence is standard practice and will be continued until 3 months after last drug intake, or agree to use a highly effective method of birth control from 28 days prior to initiation of study treatment, during treatment and until 3 months after last drug intake
Evidence of fibrosing ILD^#^ on HRCT within 12 months of visit 1 as assessed by the investigator and confirmed by central review
FVC% predicted ≥25% at visit 2^¶^
Clinically significant disease at visit 2, as assessed by the investigator based on any of the following:
Fan score ≥3 [10], or Documented evidence of clinical progression over time based on either: o 5–10% relative decline in FVC% predicted accompanied by worsening symptoms, or o a ≥10% relative decline in FVC% predicted, or o increased fibrosis on HRCT, or o other measures of clinical worsening attributed to progressive lung disease (*e.g.* increased oxygen requirement, decreased diffusion capacity)
**Exclusion criteria**
AST and/or ALT >1.5×ULN at visit 1^+^
Bilirubin >1.5×ULN at visit 1^+^
Creatinine clearance <30 mL·min^−1^ calculated by Schwartz formula at visit 1^+^
Patients with underlying chronic liver disease (Child Pugh A, B or C hepatic impairment) at visit 1
Previous treatment with nintedanib
Other investigational therapy received within 1 month or 5 half-lives (whichever is shorter but ≥1 week) prior to visit 2
Significant PAH defined by any of the following:
Previous clinical or echocardiographic evidence of significant right heart failure History of right heart catheterisation showing a cardiac index ≤2 L·min^−1^·m^−2^ PAH requiring parenteral therapy with epoprostenol/treprostinil
Other clinically significant pulmonary abnormalities (investigator-assessed)
Cardiovascular diseases (any of the following):
Severe hypertension (uncontrolled with treatment), within 6 months of visit 1; uncontrolled hypertension is defined as: o Children aged 6–≤12 years: ≥95th percentile+12 mmHg or ≥140/90 mmHg (whichever is lower) (systolic or diastolic blood pressure equal to or greater than the calculated target value) o In adolescents aged 13–17 years: systolic blood pressure ≥140 mmHg or diastolic blood pressure ≥90 mmHg Myocardial infarction within 6 months of visit 1 Unstable cardiac angina within 6 months of visit 1
Bleeding risk, defined as any of the following:
Known genetic predisposition to bleeding Patients who require: o Fibrinolysis, full-dose therapeutic anticoagulation (*e.g.* vitamin K antagonists, direct thrombin inhibitors, heparin, hirudin) o High-dose antiplatelet therapy^§^ History of haemorrhagic CNS event within 12 months of visit 1 Any of the following within 3 months of visit 1: o Haemoptysis or haematuria o Active gastrointestinal bleeding or gastrointestinal ulcers o Major injury or surgery (investigator-assessed) Any of the following coagulation parameters at visit 1: o INR >2 o Prolongation of PT by >1.5×ULN o Prolongation of aPTT by >1.5×ULN
History of thrombotic event (including stroke and transient ischaemic attack) within 12 months of visit 1
Known hypersensitivity to the trial medication or its components (*i.e.* soya lecithin)
Documented allergy to peanut or soya
Other disease that may interfere with testing procedures or in the judgement of the investigator may interfere with trial participation or may put the patient at risk when participating in this trial
Life expectancy for any concomitant disease other than ILD <2.5 years (investigator-assessed)
Female patients who are pregnant, nursing, or who plan to become pregnant while in the trial
Patients not able or willing to adhere to trial procedures, including intake of study medication
Patients with any diagnosed growth disorder such as growth hormone deficiency or any genetic disorder that is associated with short stature (*e.g.* Turner syndrome, Noonan syndrome, Russell–Silver syndrome) and/or treatment with growth hormone therapy within 6 months before visit 2^ƒ^
Patients <13.5 kg of weight at visit 1 (same threshold for male and female patients)

ALT: alanine aminotransferase; aPTT: activated partial thromboplastin time; AST: aspartate aminotransferase; CNS: central nervous system; FVC: forced vital capacity; HRCT: high-resolution computed tomography; ILD: interstitial lung disease; INR: international normalised ratio; PAH: pulmonary arterial hypertension; PT: prothrombin time; ULN: upper limit of normal. ^#^: Clinically significant fibrosing ILD will be confirmed based on documented evidence of fibrosing features on HRCT or lung biopsy, as defined in the Participants section of the Methods. ^¶^: predicted normal values will be calculated according to the Global Lung Initiative. ^+^: laboratory parameters from visit 2 will only be available after randomisation and, if the result no longer satisfies the entry criteria, the investigator will decide whether the patient should remain on study drug. Abnormal laboratory parameters at visit 1 are allowed to be re-tested (once) if it is thought that there was a measurement error or if they are a result of a temporary and reversible medical condition (once that condition has resolved). ^§^: prophylactic low-dose heparin or heparin flush as needed for maintenance of an indwelling intravenous device, as well as prophylactic use of antiplatelet therapy, are not prohibited. ^ƒ^: patients with short stature considered by the investigator to be due to glucocorticoid therapy may be included.

To match the systemic exposure reached in adult IPF, nintedanib doses (administered as soft capsules twice daily) will be based on body weight-dependent allometric scaling (scaling of adult clearance using an exponent of 0.75, consistent with the exponent estimated in population pharmacokinetic analyses in adults). A population mean nintedanib exposure of 80% to 125% compared with adult patients with IPF treated with 150 mg twice daily was targeted for the determination of planned doses by body weight in the paediatric population (Supplementary Table 1).

Dose reductions are permitted for drug-related adverse events without prior interruption, *i.e.* immediately stepping down from one dose to the next dose. If the reduced dose is well tolerated, re-escalation is possible within 4 weeks following dose reduction in cases where adverse events are considered drug related, or within 8 weeks in cases where adverse events are not considered drug related. In cases of persistent adverse events observed at the reduced dose, or severe effects at the starting dose, permanent treatment discontinuation should be considered. Temporary treatment interruption will be allowed to manage adverse events. Dose reduction and re-increase are permitted on multiple occasions.

### Ethical approval and patient consent

Trial initiation will occur at a site following review and approval by the respective institutional review board/independent ethics committee and competent authority according to national and international regulations. The trial will be conducted in accordance with the principles of the Declaration of Helsinki, International Conference on Harmonisation Guidelines, relevant sponsor standard operating procedures, and other relevant guidelines. Written informed consent and assent, where applicable, in accordance with the International Conference on Harmonisation Guidelines—Good Clinical Practice and local legislation, are required prior to trial participation.

### Randomisation and masking

Eligible patients will be randomised to treatment groups according to a randomisation plan in a 2:1 ratio (nintedanib:placebo) at visit 2 (*i.e.* the start of part A) *via* Interactive Response Technology (Almac Clinical Technologies, Souderton, PA, USA) stratified by age group (6–<12 years; 12–<18 years). Access to the codes will be controlled and documented. Validated randomisation software will be used.

### Endpoints

The primary endpoints are pharmacokinetics (area under the plasma concentration–time curve at steady state (AUC_τ,ss_) based on sampling at steady state (weeks 2 and 26) just before drug administration, and 1, 2, 3, 4, 6 and 8 h post dose) and the number (%) of patients with treatment-emergent adverse events at week 24 (table 2).

**TABLE 2:** Study endpoints

**Primary endpoints:**
Pharmacokinetics: AUC_τ,ss_ based on sampling at steady state (at week 2 and week 26)
Number (%) of patients with treatment-emergent adverse events at week 24
**Secondary endpoints:**
Number (%) of patients with treatment-emergent pathological findings of epiphyseal growth plate on imaging at week 24 and week 52^#^
Number (%) of patients with treatment-emergent pathological findings on dental examination or imaging at week 24 and week 52^#^
Number (%) of patients with treatment-emergent adverse events over the whole trial
Change in height, sitting height, leg length from baseline at week 24, week 52^#^, week 76^#^ and week 100^#^
Change in FVC% predicted from baseline at week 24 and week 52^#^
Absolute change from baseline in PedsQL at week 24 and week 52^#^
Change in *S*_pO_2__ on room air at rest from baseline at week 24 and week 52^#^
Change in 6-min walk distance from baseline at week 24 and week 52^#^
Patient acceptability based on the size of capsules at week 24
Patient acceptability based on the number of capsules at week 24
Time to first respiratory-related hospitalisation over the whole trial
Time to first acute ILD exacerbation or death over the whole trial
Time to death over the whole trial
**Further endpoints**
Number (%) of patients with increase/decrease in FVC% predicted (5–10%; >10%) at week 24 and week 52^#^
Number (%) of patients with ≥4.4-point increase in PedsQL from baseline at week 24 and week 52^#^
Number (%) of patients with >4% increase in *S*_pO_2__ on room air from baseline at week 24 and week 52^#^
Change in calculated Fan severity score from baseline at week 24 and week 52^#^
Change in HAZ score from baseline at week 24 and week 52^#^
Change in WAZ score from baseline at week 24 and week 52^#^
Slope of HAZ over whole trial
Slope of WAZ over whole trial
Number of missed school days due to the disease under study at week 24
Absolute change from baseline in log-transformed CA-125 at week 24 and week 52
**Pharmacokinetic endpoints at Visit 3 (week 2) and Visit 7 (week 26)**
*C*_max,ss_
*t*_max,ss_
*t*_1/2,ss_
CL/F_ss_
Vz/_Fss_
*C*_pre,ss_

Other parameters may be calculated as deemed appropriate. AUC_τ,ss_: area under the plasma concentration–time curve at steady state; CL/F_ss_: apparent clearance of the analyte in the plasma at steady state following extravascular multiple dose administration; *C*_max,ss_: maximum measured concentration of the analyte in plasma at steady state; *C*_pre,ss_: pre-dose concentration of the analyte in plasma at steady state immediately before administration of the next dose; FVC: forced vital capacity; HAZ: height-for-age z-score; ILD: interstitial lung disease; PedsQL: Pediatric Quality of Life Questionnaire; *S*_pO_2__: oxygen saturation measured by pulse oximetry; *t*_1/2,ss_: terminal half-life of the analyte in plasma at steady state; *t*_max,ss_: time from dosing to maximum measured concentration of the analyte in plasma at steady state; Vz/_Fss_: apparent volume of distribution during the terminal phase λz at steady state following extravascular administration; WAZ: weight-for-age z-score. ^#^: 52 weeks, 76 weeks and 100 weeks time points will not be available for all patients.

Secondary endpoints include change in FVC% predicted from baseline, Pediatric Quality of Life Questionnaire (PedsQL), oxygen saturation measured by pulse oximetry (*S*_pO_2__), and 6-min walk distance at week 24 and week 52 (table 2). Additional efficacy and safety endpoints will be collected to explore potential long-term effects (table 2).

### Key assessments

**FIGURE 3:**
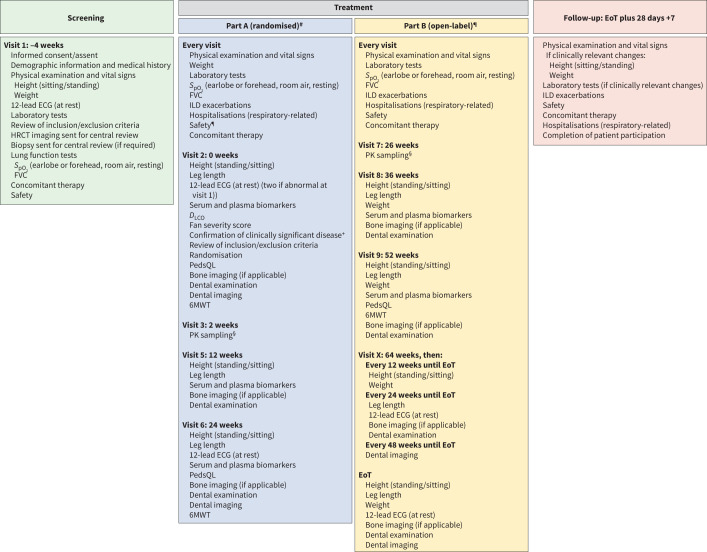
Key assessments. ^#^: Part A comprises visit 2 (week 0), visit 3 (week 2), visit 4 (week 6), visit 5 (week 12), and visit 6 (week 24). Laboratory tests can be performed between visit 5 and 6 (visit 5A) as needed. ^¶^: Part B starts at the end of visit 6 and comprises visit 7 (week 26), visit 8 (week 36), visit 9 (week 52), visit X (week 64, then every 12 weeks until end of treatment (EoT)), and EoT. Laboratory tests can be performed between each visit (visit 7A, 8A, 9A and XA as needed). ^+^: Clinically significant disease is assessed by the investigator based on any of the following: Fan score ≥3 or documented evidence of clinical progression over time (either 5–10% relative decline in forced vital capacity (FVC) % predicted accompanied by worsening symptoms, or a ≥10% relative decline in FVC% predicted, or increased fibrosis on high-resolution computed tomography (HRCT), or other measures of clinical worsening attributed to progressive lung disease (*e.g.* increased oxygen requirement, decreased diffusion capacity)). ^§^: The primary endpoints are pharmacokinetics (area under the plasma concentration–time curve at steady state (AUC_τ,ss_)) based on sampling at steady state (weeks 2 and 26) and number (%) of patients with treatment-emergent adverse events (week 24). 6MWT: 6-minute walk test; *D*_LCO_: diffusing capacity of the lung for carbon monoxide; ECG: electrocardiogram; ILD: interstitial lung disease; PedsQL: Pediatric Quality of Life Questionnaire; PK: pharmacokinetics; *S*_pO_2__: oxygen saturation measured by pulse oximetry.

Figure 3 shows key assessments. A complete physical examination and electrocardiogram will be performed at specific time points throughout the study. Potential bone toxicity will be monitored using serial assessment of leg length, height and imaging of growth plates in those patients with open physes. A routine dental examination with imaging will be carried out to monitor potential dental toxicity.

Adverse events will be coded using the Medical Dictionary for Drug Regulatory Activities, and all adverse events with an onset between start of treatment and 28 days after the last dose of trial medication will be assigned to the on-treatment period for evaluation. For the primary safety analysis, treatment-emergent adverse events during the double-blind period until week 24 (part A) will be included. A separate analysis over the whole trial period including all treatment-emergent adverse events will be performed. All treated patients will be included in the safety analysis.

While the study is not powered to detect treatment differences in efficacy outcomes like changes in FVC% predicted from baseline, efficacy assessments are planned to elucidate disease course and potential trends for treatment effect. FVC will be assessed using standardised spirometry equipment (ERT SpiroSphere) [32], with predicted values calculated according to the Global Lung Initiative [33]. *S*_pO_2__ will be measured with room air at rest by standard pulse oximetry (earlobe or forehead). Exercise capacity will be assessed using the 6-min walk test. Health-related quality of life will be assessed using PedsQL [34]. Time to first acute exacerbation (details provided in the Supplementary Methods), respiratory-related hospitalisation and time to death (all-cause mortality) will be documented.

### Additional assessments

Serum biomarker samples will be collected and submitted to the central laboratory (visits 2, 5, 6, 8 and 9 only). In selected sites, patients will have the option to participate in a longitudinal HRCT sub-study. HRCT scans will be performed at baseline, 52 weeks and 100 weeks, to identify potential predictors of progression, evaluate the association between HRCT-derived imaging and clinical parameters, and investigate computer-aided analysis for the characterisation and monitoring of chILD.

### Statistical analysis

Target sample size is based on the sample size estimation for the evaluation of the primary endpoint of pharmacokinetics and trial feasibility evaluation. For the primary evaluation of pharmacokinetics, the clearance parameter needs to be estimated with adequate precision. Assuming variability of the clearance parameter is comparable between children and adults, at least 20 patients with available pharmacokinetic measurements per age group (6–<12 years; 12–<18 years) are needed to achieve at least 80% probability (loosely referred to as power in this context [35]) of having the 95% confidence interval of apparent clearance of nintedanib in the plasma at steady state following extravascular multiple-dose administration (CL/F_ss_) and with this AUC_τ,ss_ within 60% and 140% of the geometric mean estimate, calculated as described by Wang *et al*. [35]. Pharmacokinetic assessment will be performed using non-compartmental analyses and population pharmacokinetic analyses exploring relevant covariates on pharmacokinetics (age and body weight).

An external safety monitoring committee will advise the study team and may recommend intermediate checks in those patients who switch from placebo to nintedanib at the end of the initial 24 weeks of treatment. An independent disease review committee will evaluate inclusion criteria of all screened participants, retrospectively, while an independent adjudication committee will review all fatal cases and adjudicate all deaths to either cardiac, respiratory or other causes, and review all adverse events categorised as major adverse cardiovascular events.

Safety analyses will be descriptive. As this is an exploratory study, with no confirmatory testing, analysis of secondary endpoints will be descriptive and no adjustment for multiple testing will be performed. Continuous endpoints will be analysed using a mixed model with repeated measurements. Time-to-event endpoints will be displayed descriptively using the Kaplan–Meier method. Categorical endpoints, safety and tolerability will be displayed descriptively in frequency tables.

## Discussion

This is the first randomised controlled trial of an antifibrotic agent in chILD, a group of disorders that are currently managed with limited, mainly supportive treatments [1]. Results will inform both the dose-exposure and safety profile of nintedanib in children aged ≥6 years.

Development of this study protocol required international collaboration to create a clinical trial framework in a disease area with no previous clinical trials. Unique challenges with the trial design include uncertainty regarding: 1) the prevalence of fibrotic ILD in children; 2) numbers of patients who will meet specific eligibility criteria; 3) the natural history of fibrosing ILD in children; 4) variable chILD clinical practice patterns worldwide; 5) limitations in validation of outcome measures; 6) considerations of safety and efficacy assessments when performing a study in children with ongoing lung and somatic growth. These factors impacted the approach to the study protocol as intense resource allocation was required to prepare for the implementation and standardisation of sites across a large number of countries due to the low prevalence of fibrotic ILD in children.

Similar to trials in adult fibrosing ILD [36, 37], a basket approach is being used to group children and adolescents according to demonstrated evidence of lung fibrosis and clinical disease severity, irrespective of the underlying clinical diagnosis. Hence, a major limitation of this study is the heterogeneous study population, especially in evaluating efficacy outcomes including lung function. An extensive characterisation of the patient population is needed given the differences in physiology, risk and outcome of ILD between adults and children.

Nintedanib inhibits several growth factors that are implicated in the development of IPF and other fibrosing ILDs [13–15] but may be important for lung development, *e.g.* VEGF [38]. Although most alveolarisation occurs by age 2 years [39], lung function increases throughout childhood and adolescence, and it is unclear how nintedanib may affect this process. Based on limited evidence to support the potential benefit *versus* risk of nintedanib in the growing lung and possible additional risks, including tooth development and the difficulty with assessing eligibility criteria, patients aged <6 years will be excluded from this study.

The minimum target of 30 patients and treatment duration of 24 weeks allow for adequate assessment of both systemic exposure and the tolerability profile of nintedanib in the target population. The planned dosing regimen aims to achieve nintedanib exposures in paediatric patients “similar” to those in adults (exposure-matching). The same dose/exposure across different ILDs was effective, supporting the use of the same exposure in chILD. This approach was chosen based on preclinical evidence that demonstrated antifibrotic activity of nintedanib at similar doses in several animal models of lung fibrosis [15]. Prediction of adverse events and estimation of treatment effects are complicated by limited data on the natural history of chILD. A placebo group may allow exploratory evaluation of the natural course of lung function and assessment of the background of adverse events in this patient population.

Nonetheless, evaluating potential clinical benefit in this paediatric subset is challenging. As children grow, increases in lung volume result in increased FVC, and there is the potential that FVC will increase in children with fibrosing ILD treated with nintedanib. This is in comparison with trials of fibrosing ILD in adults, where efficacy was demonstrated based on a decrease in FVC decline [25]. The benefits of improving lung structure and helping to achieve maximum lung function prior to reaching 18–21 years of age (before lung function decline begins) may have critical effects on morbidity and mortality. To support the extrapolation of the nintedanib treatment effect from adults to children, an assessment of whether data from clinical trials with nintedanib in adults can be used for the evaluation of the treatment effect in this paediatric trial (*e.g.* by incorporating the treatment effect of nintedanib in adult patients with ILDs as prior information using a Bayesian approach) is planned.

While the safety profile in chILD is presumed to be similar to that observed in the adult studies of IPF, SSc-ILD and progressive fibrosing ILDs [23–25], there are no data on nintedanib use in children. To maintain patient safety, adverse event monitoring includes guidelines for management of diarrhoea and liver function test abnormalities, imaging and clinic exams to monitor potential bone and dental toxicity, as well as an unblinded safety monitoring committee. Preclinical data [22] and clinical data from other VEGF inhibitors suggest that any potential effects on bones will be reversible with drug discontinuation. The dental toxicity noted in previous rodent studies of nintedanib has not been replicated in primate models [22], nor seen with other VEGF inhibitors in children. However, dental monitoring will be implemented to allow for early detection of any potential effects. The placebo arm will assist in the interpretation of any unexpected findings (positive or negative) in this paediatric population.

Given the high unmet medical need, lack of therapeutic options and the potential for a robust assessment of efficacy, this study design has required new thinking around the definitions and outcome measures of pulmonary fibrosis in children. In addition to providing data about the use and safety of nintedanib in children and adolescents with fibrosing ILD, the results of this trial will contribute to our understanding of the natural history and characterisation of lung impairment. The experience gained from this study will inform future interventional studies of rare paediatric diseases.

## Supplementary material

10.1183/23120541.00805-2020.Supp1 **Please note:** supplementary material is not edited by the Editorial Office, and is uploaded as it has been supplied by the author.
Supplementary material 00805-2020.SUPPLEMENT
