# Supplementary material for: Study design of a randomised, placebo-controlled trial of nintedanib in children and adolescents with fibrosing interstitial lung disease
Source: ERJ Open Res. 2021 Jun 21;7(2):00805-2020. doi: 10.1183/23120541.00805-2020 (PMC8215331; doi:10.1183/23120541.00805-2020)
Supplement: Supplementary file 1 [file 00805-2020.SUPPLEMENT.pdf]

Supplementary materials

Title: Study design of a randomised, placebo-controlled trial of nintedanib in children and adolescents with fibrosing interstitial lung disease

Authors: Robin Deterding, MD,[1,2]* Matthias Griese, MD,[3]* Gail Deutsch, MD,[4,5] David Warburton, MD,[6,7] Emily M. DeBoer, MD,[1,2] Steven Cunningham, MBChB,[8] Annick Clement, MD,[9] Nicolaus Schwerk, MD,[10] Kevin R. Flaherty, MD,[11] Kevin K. Brown, MD,[12] Florian Voss, PhD,[13] Ulrike Schmid, PhD,[13] Rozsa Schlenker-Herceg, MD,[14] Daniela Verri, PhD,[15] Mihaela Dumistracel, MD,[13] Marilisa Schiwek, MEng,[13] Susanne Stowasser, MD,[16] Kay Tetzlaff, MD,[16] Emmanuelle Clerisme-Beaty, MD,[16] Lisa R. Young, MD[17*]

Methods

Inclusion criteria for fibrosing ILD

For patients with previous pathological findings of fibrosis on lung biopsy, confirmation of fibrosis on high-resolution computed tomography (HRCT) will be made if at least one of the following imaging criteria are met within 12 months of screening (Visit 1), as confirmed by central review: reticular abnormality or traction bronchiectasis or architectural distortion (with or without ground-glass opacification) or honeycombing. Coexisting cystic abnormalities or ground-glass opacities are acceptable; however, coexisting multifocal non-fibrotic, non-dependent consolidations (e.g. organising pneumonia, infection) will not be permitted.

Any of the following lung biopsy findings or diagnoses will be accepted as documentation of fibrosis, as confirmed by central review: non-specific interstitial pneumonia (fibrosing); usual interstitial pneumonia; evidence of interstitial fibrosis on a significant component of the lung biopsy (based on the opinion of the central reviewer); evidence of lobular remodelling on a significant component of the lung biopsy (based on the opinion of the central reviewer); or honeycomb lung. For patients without any documented lung biopsy or whose biopsy results do not meet the biopsy criteria for fibrosis listed above, at least two of the aforementioned imaging findings are required on at least two HRCT scans.

Definition of acute exacerbation in chILD

Acute exacerbation will be defined as a significant worsening of the patient's respiratory condition that necessitates a change in regular management, based on >2 of the following criteria over 4 weeks: increase in respiratory rate ≥20% from baseline; increase in or development of dyspnoea; newly developing or increased abnormalities on chest imaging; onset/increase of oxygen demand to attain the individual baseline saturation (at rest and/or during exercise); need for an additional level of ventilatory support (in addition to oxygen); decrease in vital capacity ≥10% from baseline in children able to perform the test; or reduced exercise tolerance in children able to perform the tests (includes desaturation) [12].

Supplementary Table 1: Dose assignment and dose reduction based on patient body weight

Body weight bin	Weight range (kg)	Dose (BID)	Capsule strength (number required)	Dose reduction allowances (BID)	Capsule strength (number required)
1	13.5* to <23.0	50 mg	25 mg (2)	25 mg	25 mg (1)
2	23.0 to <33.5	75 mg	25 mg (3)	50 mg	25 mg (2)
3	33.5 to <57.5	100 mg	100 mg (1) or 25 mg (4)	75 mg	25 mg (3)
4	≥57.5	150 mg	150 mg (1) or 25 mg (6)	100 mg	100 mg (1) or 25 mg (4)

* Patients <13.5 kg of weight will be excluded from the trial.

BID, twice daily.
